# Comparing Working Memory and Verbal Learning in Older Adult Musicians and Non-musicians

**DOI:** 10.1093/geroni/igab046.2620

**Published:** 2021-12-17

**Authors:** Caroline Galloway, Jessica Strong

**Affiliations:** 1 University of Prince Edward Island, University of Prince Edward Island, Prince Edward Island, Canada; 2 University of Prince Edward Island, Charlottetown, Prince Edward Island, Canada

## Abstract

Previous research suggests that learning or playing an instrument may benefit working memory and executive functioning. The literature also suggests vocal training or singing ability may increase proficiency in verbal learning and working memory. Despite the benefits of musical training, the underlying mechanisms remain unclear. Older adult participants (N=38, Mean age =70.2) provided their music training history and completed a cognitive test battery. Musicians were either instrumentalists and/or vocalists (N=24) or non-musicians (N=14). Independent t test analyses were run with the current modest sample size to compare scores in basic and complex attention and working memory (Digit Span Forward (DSF) and Digit Span Backwards (DSB, and Digit Span Sequencing (DSS)), and verbal learning and memory (California Verbal Learning Test-3 (CVLT)). Results found that musicians/singers had higher scores compared to non-musicians on DSS (t(32)= -1.96, Cohen’s d =.72, p =.058) and on CVLT delayed raw scores(t(32)= -1.98, Cohen’s d=.71, p=.056), both with a medium-large effect size. There were no significant differences found between musicians and non-musicians in DSF and DSB or on CVLT immediate recall/learning. The results suggest that musical training, either instrumental or vocal, may contribute to working memory and verbal memory in older adults. Both the Digit Span task and CVLT rely heavily on executive functioning ability, which may act as a mechanism or mediator between instrumental and vocal training and scores on these cognitive tasks.

